# One Health and Gender Oncology: An Integrated Framework for Equitable Oncology Care Across the Human–Animal–Environment Interfaces

**DOI:** 10.3390/cancers18142290

**Published:** 2026-07-16

**Authors:** Rossana Berardi, Francesca Rossi, Roberto Papa

**Affiliations:** 1 Department of Oncology, Università Politecnica delle Marche, AOU Marche, 60126 Ancona, Italy; 2 Quality, Risk Management and Health Technology Innovation Unit, Department of Staff, University Hospital of Marche Region, 60126 Ancona, Italy

## 1. Introduction

Cancer is not a monolithic disease but a constellation of conditions shaped by intersecting biological, social, and ecological forces. For decades, oncology has recognized that males exhibit higher incidence and mortality in 22 of 25 non-reproductive cancers [1]; however, this knowledge rarely influences trial design. At the same time, gender, as a social construct, structures cancer risk through occupational exposures (e.g., male-dominated industries with asbestos exposure) and systemic barriers faced by transgender individuals [2,3].

Concurrently, recent pandemics have highlighted the need to connect disciplines studying animal, human, and environment health—that is, the “One Health” concept. The One Health movement has gained momentum in oncology, revealing that cancers should be an additional key focus in the One Health approach based on factors that add to the well-documented impact of humans on the natural environment and their implications for cancer emergence. First, human activities are oncogenic to other animals, exacerbating the dynamics of oncogenesis, causing immunosuppressive disorders in wildlife with effects on host–pathogen interactions, and eventually facilitating pathogen spillovers. Second, the emergence of transmissible cancers in animal species (including humans) has the potential to accelerate biodiversity loss across ecosystems and to become pandemic. Third, translating knowledge of tumor-suppressor mechanisms found across the animal kingdom to human health offers novel insights into cancer prevention and treatment strategies [4]. Dogs develop spontaneous osteosarcomas with genomic parallels to human pediatric cases [5]; beluga whales in polluted estuaries show high rates of intestinal cancer, mirroring human industrial exposure [6]. However, One Health oncology has predominantly focused on comparative tumor biology, overlooking how gender-oriented social roles mediate exposure to environmental risk.

In this editorial, we argue that sex/gender oncology is incomplete without environmental context, and that One Health without gender analysis perpetuates inequalities. By integrating emerging evidence, we map the mechanistic pathways linking sex biology, gendered exposures, and environmental carcinogens, document gaps in care for sexual and gender minority (SGM) populations, and propose an innovative framework for clinical and policy action.

## 2. The Three Critical Dimensions of Oncological Equity

### The Biological–Social–Environmental Triad

Sex differences extend far beyond reproductive organs. Males exhibit a 40–70% higher incidence of hepatocellular carcinoma (HCC), partly due to androgen receptor signaling that promotes tumor proliferation [7], but this also reflects gendered behaviors: men experience higher aflatoxin exposure than women in agricultural settings because of gender-oriented occupational roles [8]. Crucially, these pathways intersect: in vitro models show that aflatoxin B1 metabolites bind more readily to male hepatocytes due to CYP3A4 enzyme dimorphism [9], illustrating how environmental toxins exploit sex-based biological vulnerabilities.

Immunotherapy response further reveals this complexity. Females mount stronger CD8+ T-cell responses to checkpoint inhibitors across melanoma and non-small cell lung cancer (NSCLC) [10]; however, in regions with high air pollution (PM2.5 > 35 μg/m^3^), this advantage diminishes—suggesting that environmental drivers may override sex-based immunological advantages [11]. Such interactions remain unmeasured in the vast majority of immunotherapy trials [12].

## 3. Sexual and Gender Minority Populations: A Systems Thinking Approach

Transgender and gender-diverse individuals navigate complex vulnerabilities that reflect both structural discrimination and differential environmental exposures. A recent multicenter survey (n = 1247 SGM oncology patients) found that 68% delayed screening due to anticipated discrimination, while 41% reported living in neighborhoods with high levels of industrial carcinogens but without access to environmental health resources [2]. Provider knowledge gaps further exacerbate the situation: only 12% of oncologists reported feeling “very confident” in managing interactions between hormone therapy and chemotherapy [13].

A full spectrum of gynecologic malignancies has been documented in transgender men [14]. A study investigating the risks of breast cancer, often considered part of gynecologic cancer care, in transgender individuals utilizing hormone therapy found a decreased risk in transgender men compared with cisgender men and cisgender women, and a significantly increased risk of breast cancer in transgender women (usually ER/PR+) compared to cisgender men, but decreased risk when compared to cisgender women [15]. The study concluded that breast cancer screening guidelines used for cisgender individuals are sufficient for transgender people utilizing hormone therapy and is consistent with professional societal guidelines. Transgender men who have undergone mastectomy no longer require mammograms; however, there may still be value in regular self-breast exams as breast cancer has still been reported in this setting [16,17,18,19]. Taking these findings into consideration, cancer screening recommendations for transgender men are consistent with the general population; however, comparative data reveal sharp contrasts. While breast cancer screening rates in cisgender women exceed 70% in high-income countries, rates in transgender men stand at around 28% [3].

More effort is needed to understand the prevalence, as well as cancer outcomes, in these patients. Cancer surveillance studies among the transgender population will be beneficial to identify specific clinical needs and potential needs for enhanced or earlier screening for gynecologic cancers.

## 4. One Health Blind Spots: When Environmental Surveillance Ignores Gender

One Health initiatives excel at tracking zoonotic carcinogens but rarely stratify exposure data by gender. In arsenic-contaminated aquifers in Bangladesh, gender-specific physiological and behavioral factors lead to differentiated arsenic intake and health effects [20].

Similarly, veterinary oncology studies demonstrate that studying the environmental factors of cancers in dogs is paramount for advancing our understanding of cancer etiology, facilitating early detection and prevention strategies, accelerating translational research and ultimately promoting the health and well-being of all species living in a shared environment. At the same time, they highlighted the limitations that must be addressed in any oncoepidemiological study, such as inaccurate or biased information provided by owners, differences in the interpretation of questions, the inclusion of adequate covariates, the geographic location of cases (urban vs. rural vs. semirural), the direct measurement of chemical exposure, and different methodologies for case and control recruitment [21], factors in which the sex and gender of the owners can certainly play a role.

## 5. The Workforce Equity Paradox

Gender inequality permeates oncology itself, influencing research priorities. Women constitute 52% of early-career oncologists but hold only 18% of department chair positions [22]. This leadership gap correlates with research neglect: studies led by women are 3.2 times more likely to include sex-stratified analyses [12], yet they receive 17% less funding for innovative projects [23]. The consequence? Critical questions—such as how climate-driven vector expansion alters sex-specific patterns of infection-associated cancers—remain unexplored.

## 6. Toward an Integrative Framework: The Gender-Responsive One Health Model

We propose a new framework that positions gender analysis as the connective tissue linking the domains of human, animal, and environmental health. Unlike linear One Health models, our triad recognizes feedback loops: gendered occupational roles → differential environmental exposures → sex-modulated biological responses → gendered access to healthcare → unequal outcomes.

For example, there are significant disparities in the prevalence and level of occupational exposure to carcinogens among female and male workers in the Italian workforce. Moreover, in certain occupational settings women, compared to men, were more likely to be exposed to high levels of carcinogens. The overall findings provide useful information both for decision-making in prevention policies and for programming epidemiological studies on occupational cancer in the female workforce. Likewise, an accurate carcinogenic risk assessment based on concentration levels and co-exposure patterns can help to address prevention and health promotion plans in the workplace [24].

## 7. Clinical and Policy Imperatives

Three evidence-based actions emerge as priorities:

- Mandate sex/gender stratification in clinical trial design: regulatory agencies should require the reporting of sex-stratified efficacy and toxicity outcomes as a condition for approval [25];
- Co-design inclusive care pathways for SGM populations: collaborate with SGM communities to redesign clinical spaces (e.g., gender-neutral screening rooms), train providers through standardized curricula, and integrate sexual orientation and gender identity (SOGI) data into electronic health records with appropriate privacy safeguards [25];
- Integrate gender variables into One Health surveillance: environmental monitoring programs must collect gender-disaggregated exposure data and model interactions with sex-based biological susceptibility [26].

## 8. Conclusions

Oncological equity requires that we transcend siloed approaches. Sex differences in tumor biology cannot be separated from gendered environmental exposures; One Health surveillance loses relevance when it ignores how social constructions shape vulnerability. The integration we propose—a gender-responsive One Health oncology—is not merely additive but transformative: it redefines cancer as a syndemic condition emerging at the intersection of chromosomes, culture, and ecosystems.

As climate change amplifies the distribution of environmental carcinogens, this integration becomes increasingly urgent. Rising temperatures expand habitats for oncogenic vectors (e.g., Schistosoma hematobium), while extreme weather events displace communities into carcinogen-contaminated areas—disproportionately affecting gender-marginalized groups. Only by weaving sex, gender, and environmental analysis into a single framework can oncology fulfill its promise of equitable care for all bodies on a changing planet.

## Data Availability

Data sharing is not applicable to this article, as no datasets were generated or analyzed during the study.
